# Defocus Incorporated Multiple Segments (DIMS) spectacle lenses in UK children: Outcomes from a 2‐year multi‐site interventional trial

**DOI:** 10.1111/opo.70034

**Published:** 2025-10-28

**Authors:** Sara McCullough, Holly Barr, Jane Fulton, Susie Jones, Nicola Logan, Manbir Nagra, Shahina Pardhan, Patrick Richardson, Kathryn Saunders, Yasmin Whayeb, Peter Williamson, Petri Eskola, Natalia Vlasak

**Affiliations:** ^1^ Centre for Optometry and Vision Science Ulster University Coleraine UK; ^2^ Vision and Eye Research Institute, School of Medicine Anglia Ruskin University Cambridge UK; ^3^ School of Optometry Aston University Birmingham UK; ^4^ Silmäasema Oy Helsinki Finland; ^5^ Hoya Vision Care Amsterdam the Netherlands

**Keywords:** axial length, DIMS lenses, myopia, myopia control, refractive error, UK children

## Abstract

**Introduction:**

Myopia is a growing public health concern with long‐term risks for visual impairment. While Defocus Incorporated Multiple Segments (DIMS) spectacle lenses have proven efficacy in Chinese children, evidence from Western populations remains limited. This multi‐site interventional study evaluates the effectiveness of DIMS lenses in slowing myopia progression and their visual acceptability and tolerability among UK children.

**Methods:**

Children aged 5–15 years with cycloplegic spherical equivalent refraction (SER) of −0.50 to −8.50 D, anisometropia ≤1.50 D and astigmatism ≤2.50 D were recruited. All participants were prescribed DIMS spectacle lenses. SER (cycloplegic autorefraction) and axial length (AL, IOLMaster) were measured at baseline and at 6‐monthly intervals for 24 months. Measured axial elongation was compared to expected eye growth in age‐ and ethnicity‐matched untreated myopes from published meta‐analyses. Visual function (distance/near visual acuity, stereoacuity, accommodative lag and ocular posture) and visual symptoms (participant questionnaire) were also assessed.

**Results:**

A total of 108 participants completed the study to 2 years (baseline age 10.2 ± 2.2 years). Model‐adjusted mean (SE) changes in SER and AL change were −0.35 ± 0.04 D and 0.17 ± 0.01 mm at 12 months and −0.57 ± 0.05 D and 0.30 ± 0.03 mm at 24 months. Compared to expected untreated myopic progression, children wearing DIMS spectacle lenses showed 0.27 ± 0.20 mm (mean ± SD) less axial elongation over 24 months, with 91% exhibiting slower than average untreated myopic eye growth. Measures of visual function were comparable through DIMS and single‐vision spectacle lenses. Fifty‐seven percent of participants reported no visual symptoms within the first week, and on average, visual symptoms were ‘never’ or ‘seldom’ experienced by participants after 12 and 24 months of DIMS wear.

**Conclusions:**

This study provides robust and novel evidence demonstrating that DIMS spectacle lenses provide meaningful slowing of axial elongation among UK children. Minimal visual symptoms and preserved visual function throughout lens wear support their clinical viability and real‐world applicability for myopia management in diverse populations.

**Key points**

- This is the first UK‐based interventional study to evaluate Defocus Incorporated Multiple Segments (DIMS) spectacle lenses, addressing a critical evidence gap regarding their use outside East Asian populations.
- Over the 2‐year study period, DIMS spectacle lenses slowed axial elongation in the majority of children when compared with expected eye growth for age‐ and ethnicity‐matched untreated myopes.
- Visual function through DIMS spectacle lenses was comparable to single vision correction, and most children reported minimal or no visual symptoms after initial adaptation.

## INTRODUCTION

Myopia is a common refractive disorder becoming increasingly prevalent among children globally,^1^, ^2^ with the UK being no exception.^3^ Myopia has been associated with a higher risk of developing sight‐threatening conditions in later life, such as retinal detachment, glaucoma and myopic maculopathy.^4^, ^5^ Children are developing myopia at a younger age,^3^, ^6^, ^7^ and earlier onset brings scope for higher levels of myopia to ensue, further increasing the risk of sight‐threatening pathology.^8^, ^9^ With this growing concern, strategies to slow the progression of myopia have gained attention, including the application of atropine eye drops, orthokeratology contact lenses, peripheral defocus contact lenses and spectacle lenses, each demonstrating similar efficacy compared to control children wearing standard single vision lenses.^10^, ^11^, ^12^

Defocus Incorporated Multiple Segments (DIMS) spectacle lenses, commercially known as MiYOSMART (Hoya, hoyavision.com/vision‐products/miyosmartVr25/), are peripheral defocus spectacle lenses designed to slow the progression of childhood myopia. Several studies conducted in Asia^13^, ^14^, ^15^ have demonstrated the efficacy of DIMS spectacle lenses in reducing myopia progression compared to single‐vision spectacle lens correction. Further, European studies have compared DIMS lenses with other myopia management modalities and to age‐matched myopic control data,^16^, ^17^, ^18^ but there are limited data on efficacy and acceptability within a UK population. Studies report differences in the profile of myopia age of onset, prevalence and progression between ethnicities, such that East and Southeast Asian populations exhibit an earlier age of myopia onset and a faster rate of myopic progression and eye growth than their Western peers.^2^, ^7^, ^19^ Therefore, it is important to establish whether the effectiveness of myopia management strategies varies according to children's ethnicity and geographical location. It is also important to explore the acceptability of interventions across populations to ensure that children can tolerate the lens design and confirm that visual performance is not compromised. Lu et al.^20^ reported that Chinese children wearing DIMS spectacle lenses found them tolerable, with few visual complaints and minimal effect on central visual function. The most common report while wearing DIMS lenses was mid‐peripheral blurred vision and a reduction in mid‐peripheral near visual acuity, worsened by dim illumination. However, the majority (90%) of children reported a willingness to wear the DIMS spectacle lenses, with the knowledge that the lenses could slow down myopia progression.

The present single‐arm interventional study investigates the acceptance and wearability of DIMS spectacle lenses in a UK childhood cohort and the effectiveness of the spectacle lenses in slowing myopia progression and axial elongation in this cohort. A single‐arm approach to this investigation was adopted, considering the growing strength of evidence supporting the finding that the addition of peripheral defocus improves the regulation of eye growth in myopic eyes. Given the growing accessibility of myopia control interventions within the UK market at the inception of the study, recruiting and retaining participants within a control group would have been challenging and potentially unethical by withholding a treatment with established efficacy in other populations. Control groups also suffer from high dropout rates due to parental perceptions of fast myopic progression. The authors were therefore reluctant to enroll myopic children into a single vision correction ‘arm’ but rather chose to utilise data from previously published and ‘virtual’ control groups. Furthermore, the present study has purposefully included children with a wider range of ages and broader inclusion criteria for baseline spherical equivalent refraction (SER) and astigmatism than formal randomised controlled trials, to reflect better the clinical application of these interventions. The study explored the influence of adherence, age and baseline refractive error on myopic shift and axial growth.

## METHODS

### Participants and study design

This study is a prospective, multi‐site single‐arm interventional trial investigating the effectiveness and tolerability of DIMS spectacle lenses on myopic children living in the UK between April 2021 and February 2024. Informed written assent and consent were obtained from children and their parents/guardians, respectively, before commencing the study. All consenting participants were prescribed DIMS (MiYOSMART™) spectacle lenses using a subjective refraction result obtained by the researchers at baseline and dispensed according to the manufacturer's fitting guide. Subjective refraction was performed at each study visit, and study lenses were replaced if visual acuity was reduced by one logMAR line or if there was a change in spectacle lens power of ±0.50 D in either the sphere or cylinder component. Participants were instructed to wear the spectacles for all waking hours. Full details of the lens design were published previously.^13^ In summary, the DIMS spectacle lens comprises a central optical zone (9 mm in diameter) that corrects distance refractive error and an annular multiple focal zone with multiple segments (33 mm in total diameter), each having a relative positive power of +3.50 D. The design simultaneously introduces myopic defocus in the periphery but provides clear vision for the wearer through the central zone. Cycloplegic SER and axial length (AL) were measured at baseline and every 6 months for a period of 2 years. Data collection occurred at one of three UK sites:

- Anglia Ruskin University, Cambridge, England
- Aston University, Birmingham, England
- Ulster University, Coleraine, Northern Ireland

Baseline screening was performed to determine whether the child met the following inclusion criteria:

- Resident in the UK
- Age between 5 and 15 years at time of consent
- SER between −0.50 and −8.50 D in both eyes (cycloplegic autorefraction)
- Astigmatism of 2.50 D or less in both eyes (cycloplegic autorefraction)
- Anisometropia 1.50 D or less in both eyes (cycloplegic autorefraction)
- Best corrected monocular distance visual acuities of 0.20 logMAR or better in both eyes
- Willingness to wear spectacle lenses regularly

Exclusion criteria:

- Ocular or systemic abnormalities that might affect visual functions or refractive development
- Manifest strabismus
- Previous use of optical or pharmaceutical myopia control
- Previous adverse reaction to cyclopentolate or proxymetacaine eye drops

### Sample size

Myopic European children of comparable age to those required for the present study^21^ wearing single vision optical correction over a 12‐month period demonstrated an average change in AL of 0.24 ± 0.15 mm. Using DIMS spectacle lenses, Hong Kong Chinese children demonstrated 50% less axial change in a 12‐month period, compared to peers in single vision spectacle lenses.^13^ Using these data, we anticipated a change in AL of 0.12 mm in the participants within the present study. Using a power of 80% and a 5% significance level, a required sample of 53 participants was derived. To explore the impact of age (i.e., the effectiveness among children <10 years of age versus those ≥10 years of age), the sample size was doubled to 106 and inflated to 128 to allow for 20% attrition. Recruitment was distributed among the participating institutions and approximately equal numbers of children <10 and ≥10 years were screened for inclusion.

### Procedures

SER and AL were measured under cycloplegia at baseline and at 6‐monthly intervals for 2 years. Cycloplegia was induced by one drop of 1.0% cyclopentolate hydrochloride, after corneal anaesthesia using one drop of 0.5% proxymetacaine hydrochloride. Autorefraction was performed at least 20 min after the instillation of cyclopentolate hydrochloride. Confirmation of the absence of the pupillary light reflex and a lack of accommodation (<2 D) was used to confirm that cycloplegia had been achieved. A further drop of cyclopentolate was instilled if this was not the case (e.g., those with darker irides). Cycloplegic autorefraction was measured using either the Shin‐Nippon NVision‐K5001 or Grand Seiko WAM (Shin‐Nippon, rexxam.co.jp/) and axial length was measured using the IOLMaster 500 or 700 (Zeiss, zeiss.com/corporate/en/home.html). An average of at least five measurements of autorefraction and AL for each eye was used for analysis. SER was calculated as sphere + cylinder/2.

Visual functions were measured with the participant's habitual single vision spectacles (or single vision lenses in a trial frame where a significant change was identified between subjective refraction and habitual correction at the study visit) and with DIMS study spectacles at the commencement of wear. Visual function measures are included in Table 1.

**TABLE 1:** Study metrics gathered at each visit.

Data collected (method)	Baseline visit	Spectacle collection visit	1 week after spectacle collection	6 M	12 M	18 M	24 M
Best corrected distant visual acuity (ETDRS chart)^a^	X	X		X	X	X	X
Best corrected near visual acuity (HOTV chart)^a^		X		X	X	X	X
Ocular posture (prism cover test)^a^	X	X		X	X	X	X
Stereoacuity (Randot stereogram)^a^		X		X	X	X	X
Accommodative accuracy (modified Nott technique, 4D target)^a^		X		X	X	X	X
Non‐cycloplegic refractive error (subjective refraction)	X			X	X	X	X
Internal and external ocular health (slit‐lamp assessment and fundus photography)	X						
Cycloplegic refractive error (Autorefraction, ShinNippon‐NVision‐K5001 or Grand‐Seiko WAM)	X			X	X	X	X
Axial length (Biometry, IOL Master 500 or 700)	X			X	X	X	X
Visual symptoms (questionnaire)			X		X		X
Wearing schedule (questionnaire)				X	X	X	X

Abbreviations: DIMS, Defocus Incorporated Multiple Segments; ETDRS, Early Treatment of Diabetic Retinopathy Study; M, month.

^a^ Measures taken with both habitual single vision spectacles (or single vision lenses in a trial frame where a significant change was identified between subjective refraction and habitual correction at the study visit) and with DIMS study spectacles.

Visual symptoms experienced while wearing the study spectacles were collated using a questionnaire issued to the participant and their parent/guardian 1 week after collecting the study spectacles and after 12 and 24 months of DIMS spectacle wear. At 12 and 24 months, the researcher asked children to report how frequently symptoms were experienced with the study lenses using a scale ranging from 1 to 10 (‘never’ to ‘always’). Responses were grouped into ‘never’ (responses 1 or 2), ‘seldom’ (responses 3 or 4), ‘sometimes’ (responses 5 or 6), ‘often’ (responses 7 or 8) and ‘always’ (responses 9 to 10). The total symptom score represented the sum of all individual symptom frequency scores. Participants (supported by parents/guardians, as necessary) were also asked how many hours per day they wore the study spectacles during weekdays and weekends. Wearing time at each 6‐month interval was calculated using the formula: ([weekday wearing time × 5] + [weekend wearing time × 2]) ÷ 7, providing an average daily wearing time (hours per day). The overall average wearing time across the 24‐month study period was determined by averaging the daily wearing times at 6‐, 12‐, 18‐ and 24‐month study visits. Adjustments were made if the child reported removing their study spectacles or using alternative forms of optical correction.

The ethnicity of the participant was determined at baseline based on parental report using a predefined list of standardised ethnic classifications.

### Statistical analysis

Data were analysed using Intercooled Stata 13.1 software (stata.com). Central tendency measures were described using mean and standard deviations/standard errors and medians/inter‐quartile ranges. Correlations were evaluated using Linear Regression analysis where data were continuous or Spearman's correlation where data were ordinal. Differences between visual performance measures were compared using paired *t*‐tests. Myopia progression was evaluated at each visit as the difference between the cycloplegic SER at baseline and subsequent 6‐monthly visits for a period of 24 months. The change in AL was the difference between the AL at baseline and the subsequent 6‐monthly visits for 24 months. Total myopia progression and axial elongation were calculated as the difference between SER and AL at baseline to the 24‐month visit. All measurements were standardised to account for individual variations in time between visits using change in SER or AL × interval period/exact number of months between visits (e.g., change in SER over 6 months × 6/6.1 months).

An intention‐to‐treat approach was adopted, and Generalised Estimating Equations (GEE) were used to incorporate all available repeated measures data, including participants with some missing follow‐up points, thereby maximising the dataset rather than excluding incomplete cases. Baseline covariates in the model were age at enrolment, baseline SER, baseline AL, ethnicity and sex. GEE models are well suited for intention‐to‐treat repeated measures analysis as they account for the correlation between observations from the same individual, accommodate missing repeated measures and provide robust population‐averaged estimates. Average wearing time over the full study period was compared to total change in SER and AL over 24 months. Participants were subdivided into full‐time wearers if they wore the study spectacles, on average, for 12 h or more per day, or part‐time wearers who wore the study spectacles on average less than 12 h per day. Change in SER and AL over 24 months was compared between full‐ and part‐time wearers using independent *t*‐tests.

AL change data were compared to published age‐ and ethnicity‐matched data for untreated (standard correction with single vision) myopic eye growth. Brennan et al.^19^ reported that annual elongation for the subsequent year can be estimated for untreated myopic eyes using the following equation:

$$\begin{array}{c} \mathrm{Ln}\left(\text{axial elongaton}\right)=0.362-0.158\times \left(\mathrm{Age}+0.5\right)+0.325\\ \;\times \text{RaceCode} \end{array}$$

Myopic Asian children are reported to progress faster than their Non‐Asian peers, but perhaps not as rapidly as those living in East Asia.^19^ Brennan et al. assigned a RaceCode of zero for Non‐Asian children and 1.0 for those of Asian ethnicity.^19^ They recommend that a RaceCode of 0.5 be utilised for Asian children living outside of Asia, and this value was utilised for all Asian participants in the present study. Children were classified as Asian if they were of Chinese, Asian Indian, Other Asian or Mixed Race (with one Asian parent) ethnicity. All other participants were classed as Non‐Asian for the purposes of analysis.

Each participant's change in AL between baseline and 12 months and between 12 and 24 months was compared to the mean annual expected elongation for their specific age (i.e., estimated expected change in AL – measured change in AL, mm) such that positive values represent less measured annual change in AL compared to estimated annual AL change expected for an untreated age‐ and ethnicity‐matched myope. The sum of the differences in AL (expected‐measured) from baseline to 12 months and between 12 and 24 months was used to determine the cumulative difference in axial elongation over the 2‐year study period compared to age‐ and ethnicity‐matched data. Differences in change in AL between study periods (i.e., baseline to 12 months vs 12–24 months) were analysed using paired *t*‐tests.

Identifying a fully European control group from a 2‐year study to compare with the present study's outcome was challenging. The present study includes comparisons with published control groups (participants wearing standard correction with either single vision spectacles or contact lenses) from the most closely matched myopia control trials conducted with predominantly European and North American cohorts. Data from the present study were also compared to previously published randomised control trial data from children in Hong Kong (HK) wearing DIMS spectacle lenses.^13^ Sub‐group analysis was conducted to compare outcomes from participants within the present study, matched by baseline age and refractive error with those from participants in the HK trial (i.e., between 8 and 13 years, SER at baseline between −1.00 and −5.00 D, baseline cylinder ≤1.50 D). Two‐tailed, two‐sample tests of proportion and one‐sample *t*‐tests were used to compare the outcomes. A *p‐value* < 0.05 was considered statistically significant throughout.

## RESULTS

One hundred and thirty‐seven children were screened for inclusion in the study. Eight children did not meet the inclusion criteria, and one child did not like wearing the study lenses after a few days and exited the study. Of 128 children recruited to the study, 118 completed 12 months (92%) and 108 completed 24 months (81%) of the study protocol. Three participants who remained in the study at 24 months were unable to attend their 12‐month visit; therefore, complete data are available for 105 participants for both years of the study. The demographics and mean SER and AL at baseline for those who completed 12 months of the protocol and those who completed both 12‐ and 24‐month visits are presented in Table 2. There were no statistically significant differences between variables for those completing 1 versus 2 years of the study (all, *p* > 0.50).

**TABLE 2:** Demographic data and baseline spherical equivalent refraction (SER) and axial length (AL) of participants who completed the 12‐month visit and completed 12‐ and 24‐month visits.

	Completed 12‐month visit (*n* = 118)	Completed 12‐ and 24‐month visits (*n* = 105)	
Age at baseline (years)	10.3 ± 2.4 (5.4–15.7)	10.2 ± 2.2 (5.4–15.7)	*t* = −0.66, *p* = 0.51
**Sex**			
Male, % (*n*)	45% (53)	45% (47)	
Female, % (*n*)	55% (65)	55% (57)	
**Ethnicity**			
White, % (*n*)	61.9% (73)	62.5% (65)	
Asian Indian, % (*n*)	18.6% (22)	20.2% (21)	
Other Asian, % (*n*)	7.6% (9)	4.8% (5)	
Chinese, % (*n*)	6.8% (8)	6.7% (7)	
Mixed race, % (*n*)	5.1% (6)	5.8% (6)	
Baseline SER (D)	−3.16 ± 1.68 (−0.50–−7.75)	−3.11 ± 1.60 (−0.50–−7.43)	*t* = 0.35, *p* = 0.73
Baseline AL (mm)	24.65 ± 0.96 (21.84–27.30)	24.60 ± 0.93 (21.84–27.30)	*t* = −0.57, *p* = 0.57

There were no statistically significant differences between right and left eyes for baseline SER and AL nor changes in SER and AL over 12 and 24 months (all *p* > 0.50); therefore, only right eye data are presented and analysed for all variables.

### Visual performance and reported symptoms

Measures of visual functions with single vision lenses are compared with DIMS spectacle lenses (at initial collection) and are presented in Table 3.

**TABLE 3:** Mean values and standard deviations (SD) of visual function measures for participants wearing SV lenses compared with DIMS spectacle lenses (on initial collection).

	SV spectacle lenses (mean ± SD)	DIMS spectacles lenses (mean ± SD)	
Distance VA (logMAR)	−0.01 ± 0.07	−0.01 ± 0.08	*t* = −0.19, *p* = 0.85
Near VA (logMAR)	−0.03 ± 0.07	−0.02 ± 0.08	*t* = −1.22, *p* = 0.22
Near heterophoria (Δ)^a^	−1.95 ± 4.25	−1.44 ± 3.79	*t* = −2.10, *p* = 0.04
Accommodative lag (D)^b^	0.46 ± 0.38	0.47 ± 0.39	*t* = −0.29, *p* = 0.77
Stereoacuity (secs of arc)	42 ± 17	41 ± 16	*t* = 1.08, *p* = 0.28

Abbreviations: DIMS, defocus incorporated multiple segments; SV, single vision; VA, visual acuity; Δ, prism dioptre.

^a^ Positive values = esophoria, negative values = exophoria.

^b^ Positive values = accommodative lag, negative values = accommodative lead.

Near heterophoria was significantly more exophoric on average by approximately 0.5 prism dioptres (Δ) with SV spectacle lenses than when participants were corrected with DIMS spectacle lenses (*t* = −2.10, *p* = 0.04).

**FIGURE 1:**
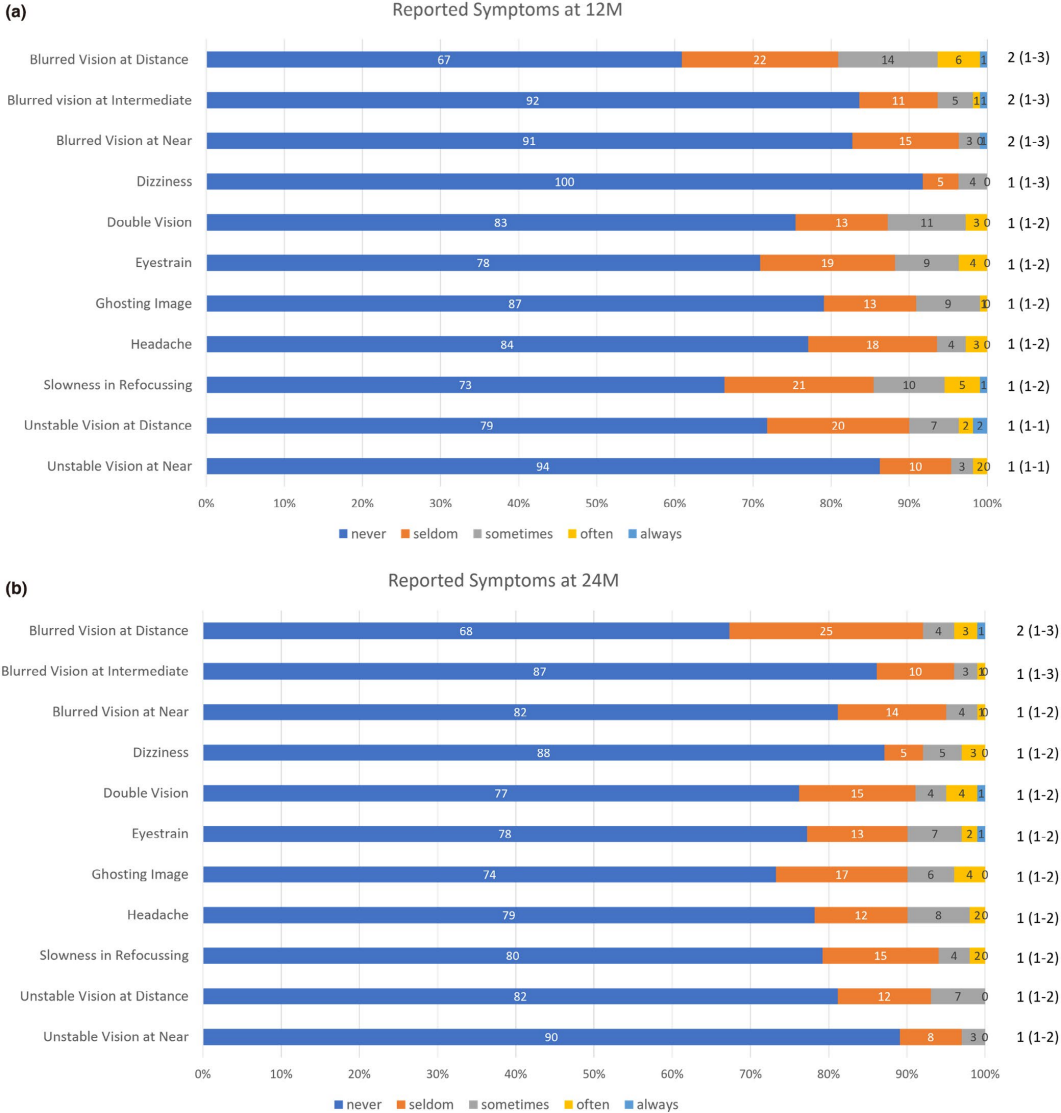
(a, b) Reported frequency of visual symptoms while wearing DIMS spectacle lenses after 12 and 24 months (M) of wear, respectively. Responses available were ‘never’ (1, 2), ‘seldom’ (3, 4), ‘sometimes’ (5, 6), ‘often’ (7, 8) or ‘always’ (9, 10). The median frequency score (Interquartile Range, IQR) for each symptom is shown on the right of the figure. The number of participants represented within each bar is labelled. DIMS, defocus incorporated multiple segments.

One child withdrew from the study within the first week, reporting dissatisfaction with the vision achieved through the lenses. Fifty‐seven per cent of children reported no visual symptoms within the first week of wearing the DIMS spectacles lenses. Figure 1a,b shows the frequency of symptoms reported while wearing DIMS spectacle lenses after 12 and 24 months of wear, respectively.

At both 12 and 24 months, the median frequency scores and interquartile ranges for all symptoms were between 1 and 3, suggesting that, on average, visual symptoms were ‘never’ or ‘seldom’ experienced by participants wearing DIMS spectacle lenses over the 2‐year study period. No child withdrew from the study due to visual symptoms associated with wearing the study spectacles.

While older children tended to report more symptoms than younger children, the correlation between participant age and total symptom score did not meet statistical significance after either 12 (Spearman's ρ = 0.18, *p* = 0.06) or 24 months of DIMS spectacle lens wear (Spearman's ρ = 0.03, *p* = 0.80). No statistically significant association was found between baseline measures of refractive error (SER, cylindrical error, anisometropia) or demographics (sex, ethnicity) and total symptom score at 12 or 24 months (all *p* > 0.11).

The most common symptom reported at both 12 and 24 months was ‘blurred distance vision’. There was no significant correlation between the frequency of reporting this symptom and the amount of change in SER between baseline and 12 months (Spearman's ρ = −0.02, *p* = 0.86), or between 12 and 24 months (Spearman's ρ = −0.03, *p* = 0.81). Nor was there a significant correlation between the measured distance visual acuity and the frequency of reporting this symptom at the 12‐ and 24‐month study visits (all *p* > 0.39).

### Change in SER and AL

#### All participants

GEE was used to account for missing data across all enrolled participants (*n* = 123) and to assess the influence of covariates on outcomes. Age at enrolment was the only covariate significantly associated with both change in SER (coefficient = 0.09, *p* < 0.0001) and change in AL (coefficient = −0.05, *p* < 0.0001) over the 24‐month study period. Other covariates included in the model (baseline SER, baseline AL, ethnicity and sex) were not statistically significantly associated with either change in SER or AL.

**FIGURE 2:**
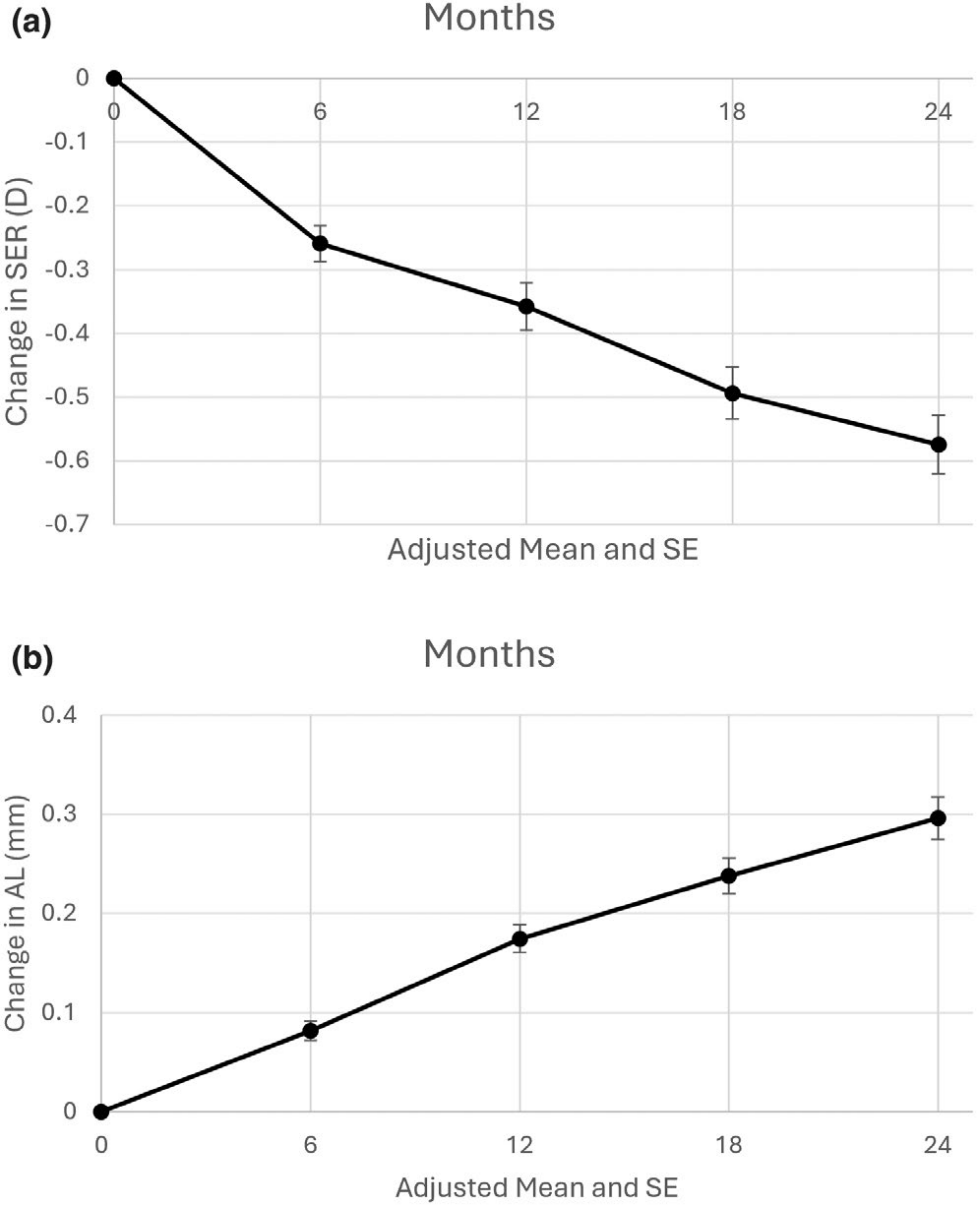
Adjusted mean and standard errors (SE) of changes in spherical equivalent refraction (a, SER, D) and axial length (b, AL, mm) from baseline to 24 months.

The model adjusted mean change in SER and AL over 24 months was −0.57 ± 0.21 D and 0.29 ± 0.12 mm, respectively. Table 4 and Figure 2a,b show the adjusted means and standard errors of the 6 monthly changes in SER and AL over the 24‐month study period.

**TABLE 4:** Adjusted mean and standard error (SE) for the 6‐monthly change in spherical equivalent refraction (SER, D) and axial length (AL, mm).

	Change in SER (D) mean ± SE	Cumulative change in SER (D) mean ± SE	Change in AL (mm) mean ± SE	Cumulative change in AL (mm) mean ± SE
6 months	−0.25 ± 0.03	−0.25 ± 0.03	0.08 ± 0.01	0.08 ± 0.01
12 months	−0.10 ± 0.04	−0.35 ± 0.04	0.09 ± 0.01	0.17 ± 0.01
18 months	−0.14 ± 0.05	−0.49 ± 0.05	0.07 ± 0.02	0.24 ± 0.02
24 months	−0.08 ± 0.05	−0.57 ± 0.05	0.06 ± 0.02	0.30 ± 0.02

#### Completed participants

The mean (±SD) change in SER and AL over 24 months for 105 participants who completed both the 12‐ and 24‐month study visits was −0.59 ± 0.57 D and 0.30 ± 0.28 mm, respectively, demonstrating that controlling for significant covariates and missing data had little effect on the unadjusted means.

### Effect of wearing time

Most children reported wearing the DIMS study spectacles all waking hours, and the majority (87%) were classed as full‐time wearers (≥12 h per day). Some children reported removing the study spectacles occasionally to wear single vision contact lenses or goggles for sport. Average wearing time was 13.0 ± 1.8 h (range 6–15 h) per day. There was a statistically significant correlation between the age of the participant and the average wearing time (*r* = 0.22, *p* = 0.02); therefore, age was controlled for in regression analysis. There was no statistically significant correlation between wearing time and change in SER (coefficient = −0.29, *p* = 0.40) or AL (coefficient = −1.40, *p* = 0.08) over the 24‐month study period. While children wearing the study spectacles full‐time showed less change in SER (−0.58 vs −0.67 D) and AL (0.29 vs 0.43 mm), on average, compared to part‐time wearers, this difference did not reach statistical significance for either parameter (both *p* > 0.07).

### Comparison to age‐ and ethnicity‐matched untreated myopes

**FIGURE 3:**
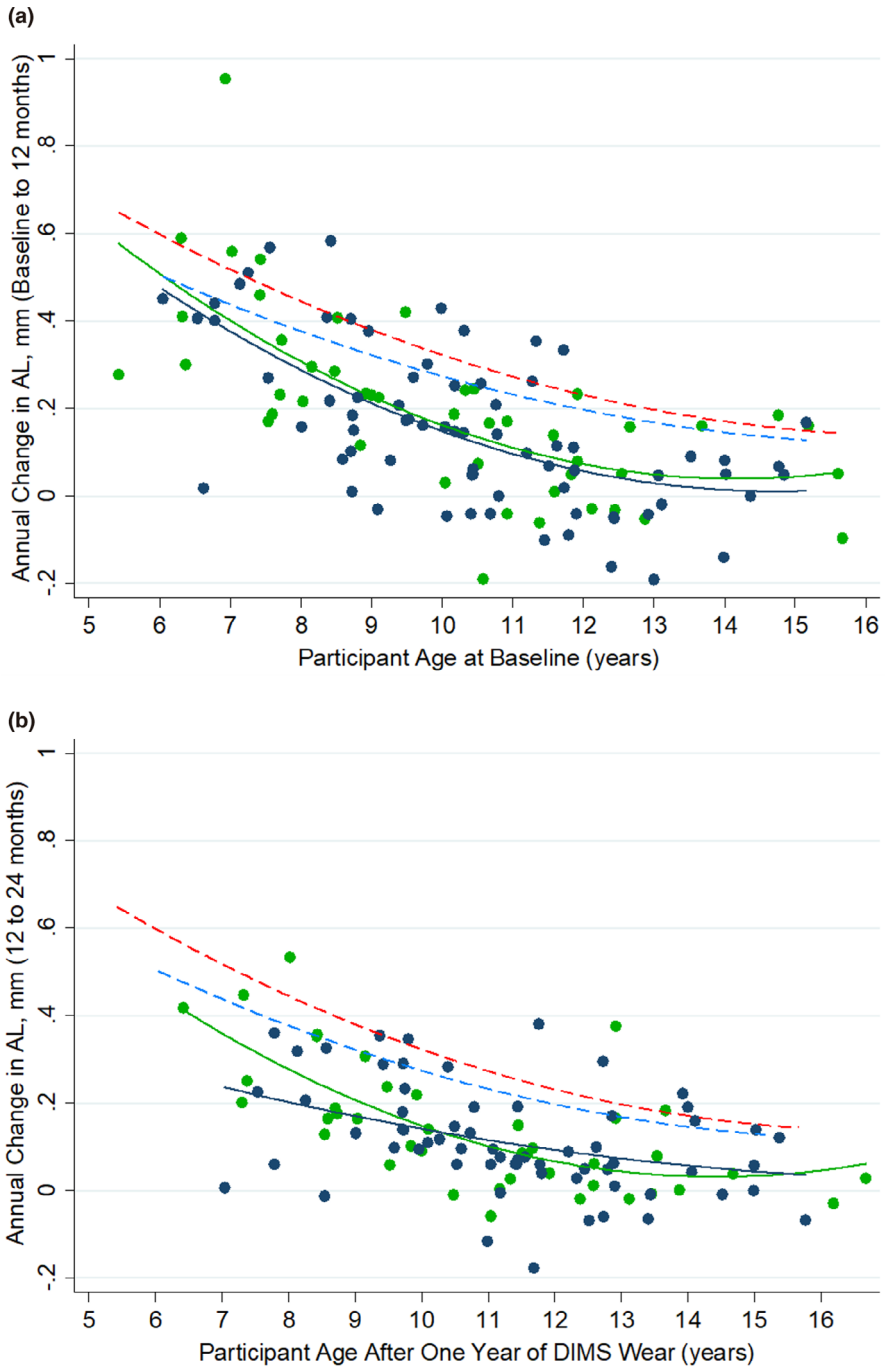
(a, b) Annual change in axial length (AL) of Defocus Incorporated Multiple Segments (DIMS) spectacle lens wearers between baseline and 12 months (a) and between 12‐ and 24‐months (b) compared to the age of the child at each visit (Navy dots = Non‐Asian, Green dots = Asian). Mean fitted annual change in AL for DIMS spectacle lens wearers (Navy line = Non‐Asian, Green line = Asian) and mean fitted expected age‐ and ethnicity‐matched annual change in AL if the child was an untreated myope (blue dashed line = Non‐Asian, red dashed line = UK Asian, derived for each participant from the Brennan et al.^19^ formula).

Figure 3a,b plot each participant's annual change in axial length between baseline and 12 months and between 12 and 24 months against age. Brennan et al.'s^19^ formula was used to plot the expected annual axial elongation of Asian and Non‐Asian untreated myopic eyes by age.

Table 5 presents the expected change in AL, based on participants' age and ethnicity, if they were untreated myopes, compared with their measured change in AL while wearing DIMS spectacle lenses. On average, the participants exhibited 0.27 ± 0.20 mm less change in AL over the 24‐month study period when wearing DIMS spectacle lenses compared to expected eye growth for untreated myopes. The difference between measured and expected AL change was of a similar magnitude in both the first and second years of lens wear (*t* = −0.49, *p* = 0.63).

**TABLE 5:** Comparison of measured change in axial length (AL) for Defocus Incorporated Multiple Segments (DIMS) spectacle lens wearers versus their expected change in AL as untreated myopes by age.

	Expected change in AL for untreated myope by age and ethnicity, mm mean ± SD (range)	Measured change in AL with DIMS wear, mm mean ± SD (range)	Difference, mm mean ± SD (range)	AL change less than untreated myopes (*n*/*N*, %)	
Baseline to 12 months	0.30 ± 0.12 (0.12–0.66)	0.17 ± 0.19 (−0.19 to 0.59)	0.13 ± 0.15 (−0.43 to 0.48)	95/118, 81%	*t* = −0.49, *p* = 0.63
12–24 months	0.26 ± 0.10 (0.11–0.57)	0.13 ± 0.13 (−0.18 to 0.53)	0.13 ± 0.11 (−0.17 to 0.43)	91/105, 87%	
Baseline to 24 months	0.56 ± 0.21 (0.24–1.23)	0.29 ± 0.28 (−0.23 to 1.09)	0.27 ± 0.20 (−0.28 to 0.67)	96/105, 91%	

*Note*: Positive values = less change in AL in DIMS wear versus untreated myopes; negative values = more change in AL in DIMS wear versus untreated myopes. Paired *t*‐test compared differences between the first and second years of lens wear.

### Comparison with European and North American control groups

Table 6 presents baseline, 12‐ and 24‐month change in SER and AL data for participants in the present study wearing DIMS spectacle lenses, alongside control groups from a range of myopia control trials using standard SV spectacle or contact lens correction in predominantly European and North American cohorts. The children within the present study showed significantly less change in SER and AL than all cohorts apart from the Myopia Outcome Study of Atropine in Children (MOSAIC) trial control group^22^ for SER over 12‐ and 24‐months and the Clinical Evaluation of MyoCare in Europe (CEME) trial for SER over 12 months.^23^

**TABLE 6:** Baseline, 12‐ and 24‐month change in spherical equivalent refraction (SER) and axial length (AL) for participants within the present study (DIMS UK) wearing Defocus Incorporated Multiple Segments spectacle lenses (DIMS SL, hoyavision.com/vision‐products/miyosmartVr25/) alongside control arms (either wearing single vision spectacle or contact lenses) in randomised control trials (RCT).

	DIMS UK	MiSight RCT controls; Chamberlain et al.^21^	CHAMP RCT controls; Zadnik et al.^24^	CYPRESS RCT controls; Rappon et al.^25^; Laughton et al.^26^	MOSAIC RCT controls; Loughman et al.^22^	Nucci et al.^16^ controls	CEME RCT controls; Alvarez‐Peregrina et al.^23^	Lawrenson et al.^11^ controls	Mean
*n*	105	74	165	93	66	32	117	>3000	
Study sites	United Kingdom	Portugal, United Kingdom, Singapore, Canada	North America, Europe	North America	Ireland	Italy	Spain, Portugal	Global	
Intervention arm	DIMS SL	MiSight CL	LDA	DOT SL	LDA	DIMS SL, LDA, DIMS SL + LDA	MyoCare SL	Mixed	
Baseline age	10.2 ± 2.2	10.1 ± 1.4	8.8 ± 1.8	8.2 ± 1.2	13.7 ± 2.2	11.3 ± 4.0	10.0 ± 1.9	n/a	
**SER (D)**									
Baseline	−3.11 ± 1.60	−2.19 ± 0.81	−2.45 ± 1.13	−1.95 ± 1.02	−3.98 (−4.69, −2.60)	−1.54 ± 0.74	−2.12 ± 0.94	n/a	
12‐month change (diff)	−0.35 ± 0.45	−0.58 ± 0.41 (+0.23)^a^	−0.60 (+0.25)^a^	−0.53 ± 0.46 (+0.18)^a^	−0.23 (−0.12)^b^	−0.85 (+0.50)^a^	−0.41 ± 0.41 (+0.06)^c^	−0.65 (+0.30)^a^	+0.23
24‐month change (diff)	−0.59 ± 0.57	−0.92 ± 0.53 (+0.33)^a^	−1.00 (+0.41)^a^	−0.88 ± 0.77 (+0.29)^a^	−0.63 ± 0.62 (+0.04)^c^	n/a	n/a	−1.01 (+0.42)^a^	+0.30
**AL (mm)**									
Baseline	24.60 ± 0.93	24.46 ± 0.70	24.33 ± 0.84	24.03 ± 0.78	25.29 ± 1.17	24.64 ± 0.79	24.17 ± 0.75	n/a	
12‐month change (diff)	0.17 ± 0.19	0.24 ± 0.15 (−0.07)^a^	0.37 (−0.20)^a^	0.30 ± 0.17 (−0.13)^a^	0.23 (−0.06)^a^	0.22 (−0.05)^a^	0.23 ± 0.15 (−0.06)^a^	0.33 (−0.16)^a^	−0.10
24‐month change (diff)	0.29 ± 0.28	0.45 ± 0.23 (−0.16)^a^	0.63 (−0.34)^a^	0.53 ± 0.33 (−0.24)^a^	0.40 ± 0.30 (−0.11)^a^	n/a	n/a	0.56 (−0.27)^a^	−0.22

*Note*: Intervention arms included (for reference only) MiSight MFSCL (multifocal soft contact lenses, missight.com), low dose atropine (LDA), Diffusion Optics Technology spectacle lenses (DOT SL, sightglassvision.com), MyoCare SL (spectacle lenses, zeiss.com) and combined treatments. Diff = difference between DIMS UK data– Control data.

^a^ Statistically significantly less change in SER or AL by DIMS UK group compared to controls (all *p* < 0.003).

^b^ Significantly greater change in SER by DIMS UK group compared to controls (*p* = 0.003).

^c^ No significant difference between DIMS UK and controls (*p* > 0.09).

### Comparison with children wearing DIMS spectacle lenses in Hong Kong

**FIGURE 4:**
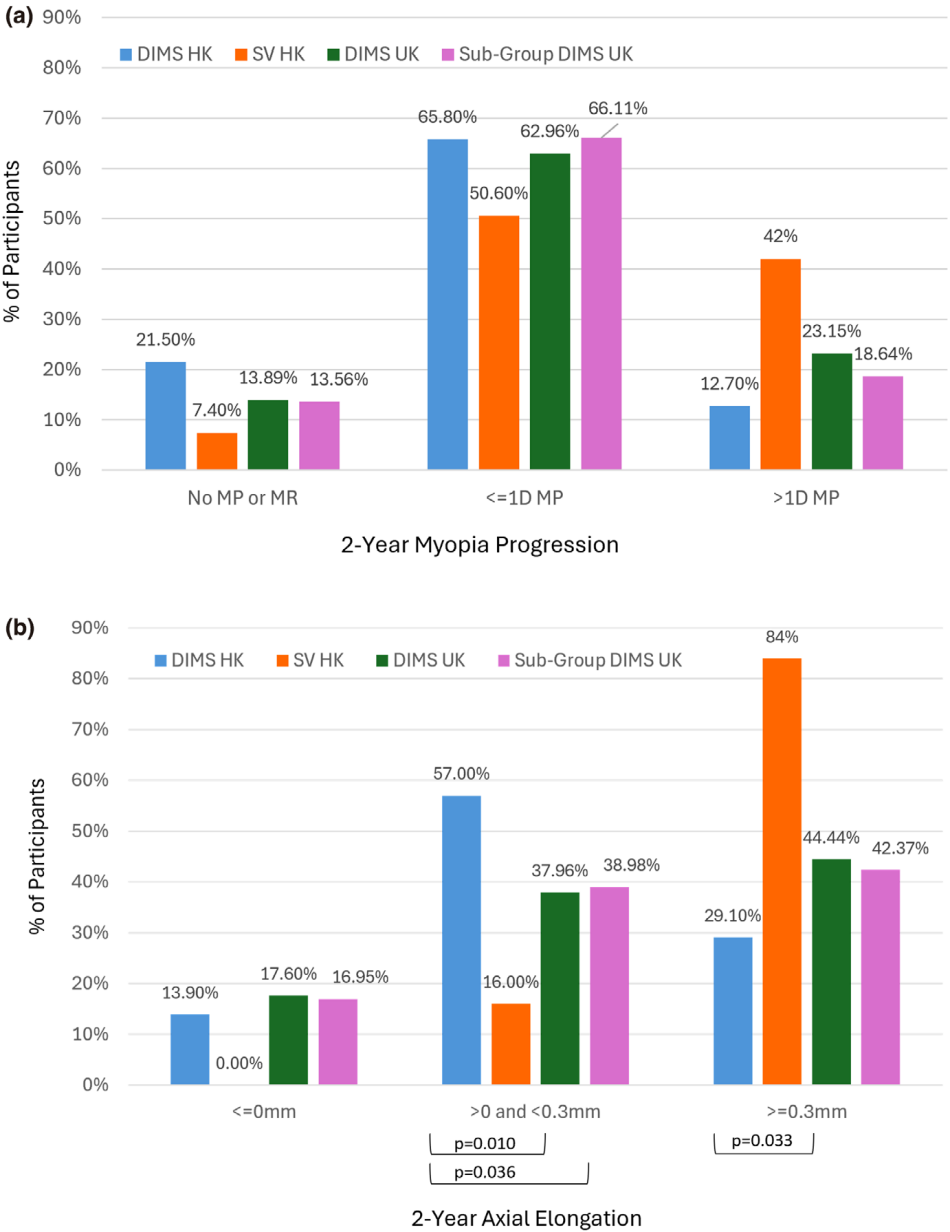
(a, b) Myopic progression (a) and axial elongation (b) over the 24‐month study period by category. MP, myopia progression; MR, myopia reduction. The presence of statistically significant *p*‐values from two sample tests of proportion between DIMS UK/Sub‐Group DIMS UK and DIMS HK is indicated on the graphs. DIMS, Defocus Incorporated Multiple Segments; HK, Hong Kong; SV, single vision. The Sub‐Group DIMS UK refers to a subgroup matched for age and baseline refractive error to the DIMS HK cohort.

Figure 4a,b present the percentage of participants categorised by pre‐defined amounts of myopic progression and axial elongation over the 24‐month study period alongside previously published findings on DIMS spectacle lens wearers from Hong Kong (DIMS HK^13^). Data from the present study are presented in terms of the total cohort (DIMS UK, *n* = 108) and a subgroup matched for age and baseline refractive error to the DIMS HK cohort (Sub‐Group DIMS UK, *n* = 59). For comparison, data from Hong Kong children wearing single vision (SV) spectacles are also included (SV HK, *n* = 81). Baseline data for the Sub‐Group DIMS UK and DIMS HK are presented in Table 7.

**TABLE 7:** Baseline demographic data, spherical equivalent refraction and axial length for participants within the Defocus Incorporated Multiple Segments (DIMS) UK Sub‐Group and the DIMS Hong Kong (HK) study.

	DIMS UK (sub‐group) *n* = 59	DIMS HK *n* = 79	
Age at baseline (years)	10.20 ± 1.32	10.20 ± 1.47	*t* = −0.02, *p* = 0.99
**Sex**			*z* = −0.67, *p* = 0.50
Male, % (*n*)	52.5 (31)	58.2 (46)	
Female, % (*n*)	47.5 (28)	41.8 (33)	
Spherical equivalent refraction (D)	−2.59 ± 1.12	−2.96 ± 0.97	*t* = 2.56, *p* = 0.01
Axial length (mm)	24.53 ± 0.65	24.70 ± 0.82	*t* = −2.08, *p* = 0.04

No statistically significant differences were found in the distribution of myopic progression categories between DIMS UK/Sub‐Group DIMS UK and DIMS HK children (all *p* > 0.07). A similar proportion of children in all DIMS groups showed no axial elongation over 24 months (all *p* > 0.50). However, significantly fewer children in the DIMS UK and Sub‐Group DIMS UK cohorts exhibited >0 to <0.3 mm of axial elongation compared with the DIMS HK group (both *p* < 0.04). A greater proportion of DIMS UK children showed axial elongation ≥0.3 mm over 24 months compared to DIMS HK children (44.4% vs 29.1%, *p* = 0.03); this difference was not statistically significant when comparing Sub‐Group DIMS UK with DIMS HK (42.4% vs 29.1%, *p* = 0.11).

Participants who demonstrated myopic progression >1 D over 2 years were significantly younger (8.5 ± 1.9 years, *t* = 4.36, *p* < 0.0001) than those showing slower myopic progression (≤1 D, 10.6 ± 2.1 years). There were no statistically significant differences in baseline SER, baseline AL, gender or ethnicity between those who showed myopic progression >1 D and those showing ≤1 D over the 2‐year period (all *p* > 0.10). Similarly, participants demonstrating axial elongation ≥0.3 mm over the 2‐year period were younger (8.6 ± 1.7 years vs 11.4 ± 1.8 years; *t* = 8.11, *p* < 0.0001) and had shorter baseline AL (24.4 ± 0.9 mm vs 24.8 ± 0.9 mm, *t* = 2.4, *p* = 0.02) than those showing slower eye growth <0.3 mm. Other demographics and baseline parameters were not statistically different between those showing faster/slower myopia progression and eye growth (all *p* > 0.17). Detailed comparisons of baseline parameters and participant demographics for the 2‐year myopia progression and axial elongation groups are available in Data S1.

Table 8 shows the changes in AL and SER from Sub‐Group DIMS UK compared with the same measures from published data on DIMS HongKong (HK). There were no statistically significant differences for the change in SER or AL between the groups at the 12‐ and 24‐month visits (one sample *t*‐tests, all *p* > 0.06).

**TABLE 8:** Mean ± standard error (SE) change in spherical equivalent refraction (SER, D) and axial length (AL, mm) at the 12‐ and 24‐month visits for the sub‐group of Defocus Incorporated Multiple Segments (DIMS) UK spectacle lens wearers from the present study and Hong Kong DIMS (DIMS HK) spectacle lens wearers.

Visit	DIMS UK (sub‐group) *n* = 59	DIMS HK *n* = 79	Mean difference	One sample *t*‐test	DIMS UK (all) *n* = 105	SV HK *n* = 81
	**Change in SER (D), mean ± SE**					
12‐month	−0.28 ± 0.06	−0.17 ± 0.05	−0.11	*t* = −0.17, *p* = 0.07	−0.35 ± 0.04	−0.55 ± 0.04
24‐month	−0.51 ± 0.07	−0.38 ± 0.06	−0.13	*t* = −0.38, *p* = 0.06	−0.57 ± 0.05	−0.93 ± 0.06
	**Change in AL (mm), mean ± SE**					
12‐month	0.14 ± 0.02	0.11 ± 0.02	0.03	*t* = 0.11, *p* = 0.16	0.17 ± 0.01	0.32 ± 0.02
24‐month	0.25 ± 0.03	0.21 ± 0.02	0.04	*t* = 0.21, *p* = 0.22	0.30 ± 0.02	0.53 ± 0.03

*Note*: Single‐vision (SV) HK and the full DIMS UK data are included for reference.

## DISCUSSION

This 2‐year single‐arm interventional study provides evidence supporting the effectiveness and tolerability of DIMS spectacle lenses in controlling myopia progression among UK children. Over 90% of children wearing the DIMS spectacle lenses showed slower than average untreated myopic eye growth for their age and ethnicity over the 2‐year trial. Compared to expected eye growth for untreated myopes, the cumulative difference in axial elongation (0.27 ± 0.20 mm) was not dissimilar to 2‐year studies of DIMS spectacle lenses in Hong Kong (Lam et al.^13^; 0.32 mm) and other optical myopia management interventions such as dual focus contact lenses (Chamberlain et al.,^21^ 0.24 mm), orthokeratology (Cho and Cheung,^27^ 0.27 mm), Highly Aspheric Lenslets (Bao et al.,^28^ 0.35 mm) and Diffusion Optics Technology lenses (Rappon et al.,^29^ 0.21 mm). While some reservations should be acknowledged regarding comparisons between myopia control studies, given that outcomes can vary considerably depending on the demographics of both the treatment and control groups, the current findings still indicate that, on average, changes in SER and AL were slower in the present study than would be expected with conventional SV correction. Compared with control groups from predominantly European and North American cohorts, DIMS spectacle lenses significantly slowed myopia progression and axial elongation in most cases, with the exception of the MOSAIC trial control group (Loughman et al.^22^), who were considerably older at baseline, which likely contributed to slower progression within their group.

From this GEE model, sex, ethnicity, baseline SER and AL did not show significant associations with the change in SER and AL. By contrast, younger age was associated with significantly greater change in SER and AL. This is not unexpected, as younger eyes are known to be growing at a faster rate than older eyes and myopia management interventions are thought to have relatively consistent impact on eye growth in absolute terms, independent of age.^30^ This notion of absolute treatment effects being relatively independent of age is supported by the present data. When compared with age‐ and ethnicity‐matched eye growth expected in untreated myopes, a comparable reduction in axial elongation was demonstrated across ages (Figure 3). The treatment effect within the current study was also relatively uniform in the first and second year of DIMS spectacle wear, in contrast to previous studies which reported greater treatment effects in the early stages of intervention and lesser effects in the subsequent years.^26^, ^30^, ^31^ The difference could be, in part, attributed to the timing of recruitment and the initial stages of the present study falling within March to December 2021, shortly after the strictest COVID‐19 confinements were lifted and during a time frame when restrictions on leisure activities continued. The effects of COVID‐19 home confinements on accelerated myopia progression are well documented,^32^ and may have potentially diminished the effectiveness in the first year of DIMS spectacle lens wear within this cohort, particularly with younger participants who have been reported to be more susceptible to myopigenic lifestyle changes.^33^ Similar findings were reported when low‐dose atropine^34^ and Diffusion Optics Technology spectacle lenses^26^ were tested during periods significantly affected by COVID‐19 restrictions.

While no statistically significant differences were found between the DIMS UK Sub‐Group and DIMS HK cohorts with respect to change in SER or AL over 12‐ and 24 months, DIMS UK participants showed slightly faster myopia progression and eye growth, contrary to expectations.^35^ This discrepancy may reflect a cohort effect, including differences in timing of data collection in the UK group or ethnic variation in relative peripheral refraction. East Asian myopes typically exhibit greater peripheral hyperopia,^36^ which may enhance the myopic defocus signal from the DIMS spectacle lenses and potentially contribute to a stronger response.^37^

### Visual symptoms and tolerability

Overall, the DIMS spectacle lenses were well tolerated by UK children. After an initial adaptation period, 57% of children reported no visual symptoms. At both 12‐ and 24 months, the median frequency scores and interquartile ranges for all symptoms were between 1 and 3, suggesting that visual symptoms were ‘never’ or ‘seldom’ experienced by most children wearing the DIMS spectacle lenses over the 2‐year study period. Blurred distance vision emerged as the most reported symptom after 12‐ and 24 months; however, reporting this symptom did not correspond with reduced measures of distance visual acuity or more rapid myopia progression. This may be due to the reliance on the child's ability to self‐report symptoms, which is likely to be influenced by individual variability in visual sensitivity and expectations. The lack of correlation may also reflect subjective perceptions of blur arising from peripheral aberrations during off‐axis viewing and activities involving dynamic eye movements or adaptation processes not captured by the standard visual performance measures used within the present study. Comparable central visual function measures were achieved with both DIMS spectacle lenses and standard single vision lenses. However, it is useful for clinicians to be cognisant of these subjective reports when prescribing DIMS spectacle lenses and to counsel neophyte DIMS lens patients appropriately regarding adaptation. A single participant withdrew from the study within the first week, reporting dissatisfaction with the vision achieved through the lenses. All other participants were willing to wear the DIMS spectacle lenses for the duration of the trial and were content with the visual performance achieved through the lenses. The level of tolerability and central visual clarity reported in the present study aligns with previous research among Chinese children,^20^ and supports the suitability of DIMS lenses as a clinically viable option for myopia management in UK children.

Tolerability of myopia control spectacle lenses is likely to be influenced by optical design and central clear zone size. DIMS lenses have a relatively large (9 mm) central clear zone surrounded by discrete +3.50 D segments. Highly aspheric lenslet technology (HALT) lenses also feature a 9 mm clear zone but create a continuous volume of peripheral defocus. Cylindrical annular refraction elements (CARE) lenses use alternating annular defocus zones around an 8–10 mm central clear zone, while Diffusion Optics Technology (DOT) lenses rely on microscopic diffusers and have a smaller central zone of approximately 5 mm. Wolffsohn et al.^38^ reported that various myopia control lens designs have minimal impact on central and peripheral vision, showing non‐inferiority to SV lenses; findings consistent with the current study. Clinical decisions regarding lens choice may be guided more by lens availability and patient preference than anticipated differences in tolerability or visual performance.

### Variability in response

A subset of participants demonstrated no myopia progression or even hyperopic shifts in SER (14%) and axial shortening (18%) over the 2 years. Nine children exhibited both outcomes. By contrast, some children showed >1.00 D of myopic progression (23%), or ≥0.3 mm of eye growth (44%) over the study period. These outcomes were more evident in younger children and those with shorter baseline AL; similar findings have been reported with DIMS spectacle lens wear in various clinical populations.^18^ Other baseline parameters and demographics were not predictive of such outcomes. Further studies are required to identify factors that predict both exceptional and poorer responses to DIMS spectacle lenses and would allow improved targeting of patients and better management of patient expectations.

### Lens‐wearing behaviour and adherence

Participants wore the study spectacles for an average of 13 ± 2 h per day, with most wearing them during all waking hours. While there was a weak positive correlation between age and wearing time (*r* = 0.22, *p* = 0.02), wearing time was not significantly associated with the change in SER or AL over 2 years. Children who wore the DIMS spectacle lenses full‐time (≥ 12 h per day) showed less change in SER and AL over the study duration, but this did not meet statistical significance for either metric. This contrasts with other studies that reported superior results with increased wearing times.^28^, ^39^, ^40^ Participants were advised to wear the DIMS spectacles during all waking hours as part of the study protocol. This finding suggests that, beyond a threshold of sufficient adherence, the effectiveness of DIMS lenses is not influenced significantly by variations in daily wear time. Younger children within the study who had shorter wear durations were also more likely to have less awake time and potentially less time experiencing myopigenic environments.

### Strengths and limitations

This study contributes novel insights into the use of DIMS spectacle lenses in a UK paediatric population, complementing existing research from East Asia and Europe in both effectiveness and acceptability of wear. The current study also extends the available data on DIMS spectacle lens wear for younger children, providing additional insight into the effect of age on treatment efficacy. While the single‐arm design does not allow for direct comparison to a control group of the same population, the recent publication of a large‐scale meta‐analysis of ethnicity‐ and age‐specific annual predicted myopic eye growth is a valuable alternative. The growing body of control data from other myopia control treatment trials also provided reference data. However, we acknowledge the limitation of not including a direct, concurrent control group within the same population. Such a group may have better accounted for lifestyle factors, educational demands, environmental influences (including the COVID‐19 restrictions) and geographical location, factors which all affect myopia progression. Nonetheless, it is important to note that even in randomised controlled trials, fully accounting for differences in lifestyle and environmental exposures between the intervention and control groups is challenging.

The a priori sample size of 106 was calculated to provide adequate power, with an initial recruitment target of 128 to accommodate potential attrition. As expected in a prospective study, some participants were lost to follow‐up. A total of 105 participants completed the study, representing a minimal shortfall that is unlikely to have meaningfully impacted statistical power. Furthermore, the analysis used generalised estimating equation (GEE) models, which accommodated missing data and reduced potential bias due to attrition. Wearing schedules were self‐evaluated and may not truly reflect actual wearing schedules; although most children wore the study spectacles all waking hours of the day with limited removal, with wear times determined by the number of hours the children were awake. Objective wear‐time sensors could offer more accurate and reliable data for this metric. Another limitation was the study's lack of investigation into potentially influential lifestyle factors, such as near‐work activities and time spent outdoors, which are known to affect myopia progression and axial elongation in both single vision wearing myopes^41^ and those undergoing myopia control interventions.^42^ Indeed, lifestyles were likely to have differed at the start of the study, compared with the later stages due to the lifting of COVID‐19 restrictions.

## CONCLUSIONS

This 2‐year single‐arm interventional study provides evidence to support the effectiveness and tolerability of DIMS spectacle lenses in managing myopia progression among UK children. The majority of participants experienced slower axial elongation than expected for untreated myopes of the same age and ethnicity, with treatment effects comparable to those reported in controlled trials and with other myopia interventions. While age influenced progression rates, there was a relatively consistent treatment effect on eye growth from DIMS spectacle lens wear in absolute terms, independent of age. DIMS lenses were generally well tolerated, with minimal visual symptoms after the adaptation period and high rates of adherence among participants. Variability in individual responses was observed, with some children showing minimal progression and others exhibiting greater than average changes, underscoring the need for further research into predictors of treatment response. Overall, these findings support the clinical utility of DIMS spectacle lenses for myopia management in a UK paediatric population.

## AUTHOR CONTRIBUTIONS

**Sara McCullough:** Data curation (lead); formal analysis (lead); funding acquisition (equal); investigation (equal); methodology (equal); project administration (lead); supervision (equal); validation (lead); visualization (lead); writing – original draft (lead). **Holly Barr:** Investigation (equal); project administration (supporting); writing – review and editing (supporting). **Jane Fulton:** Investigation (equal); project administration (supporting); writing – review and editing (equal). **Susie Jones:** Investigation (equal); project administration (supporting); writing – review and editing (supporting). **Nicola Logan:** Conceptualization (equal); funding acquisition (equal); investigation (equal); methodology (equal); project administration (supporting); resources (equal); supervision (equal); writing – review and editing (equal). **Manbir Nagra:** Conceptualization (equal); funding acquisition (equal); methodology (equal); project administration (supporting); writing – review and editing (equal). **Shahina Pardhan:** Conceptualization (equal); funding acquisition (equal); project administration (equal); resources (equal); supervision (equal); writing – review and editing (supporting). **Patrick Richardson:** Funding acquisition (equal); investigation (equal); resources (equal); writing – review and editing (supporting). **Kathryn Saunders:** Conceptualization (equal); investigation (supporting); methodology (equal); project administration (equal); resources (equal); supervision (equal); writing – review and editing (equal). **Yasmin Whayeb:** Investigation (equal); project administration (supporting); writing – review and editing (supporting). **Peter Williamson:** Investigation (equal); project administration (supporting); writing – review and editing (supporting). **Petri Eskola:** Conceptualization (equal); funding acquisition (equal); methodology (equal); writing – review and editing (equal). **Natalia Vlasak:** Conceptualization (equal); funding acquisition (equal); methodology (equal); project administration (supporting); writing – review and editing (equal).

## FUNDING INFORMATION

This study is funded by Hoya Vision Care, which included financial support to facilitate the proper conduct and delivery of the study.

## CONFLICT OF INTEREST STATEMENT

Author PE was involved in the study design and initial trial setup while employed by Hoya Vision Care and has reviewed both the initial and subsequent drafts of the manuscript. NV is currently employed by Hoya Vision Care and reviewed the initial and subsequent drafts. Neither author was involved in data collection or analysis. MN is currently employed by CooperVision International Ltd. Other authors are academic researchers who were invited to deliver the project and have no conflicts of interest to declare relevant to the submitted work.

## Supporting information


Data S1.
