# Supplementary material for: Defocus Incorporated Multiple Segments (DIMS) spectacle lenses in UK children: Outcomes from a 2‐year multi‐site interventional trial
Source: Ophthalmic Physiol Opt. 2025 Oct 28;45(7):1965–80. doi: 10.1111/opo.70034 (PMC12682106; doi:10.1111/opo.70034)
Supplement: Supplementary file 1 — Data S1. [file OPO-45-1965-s001.docx]

|  | **2-Year Myopia Progression** | | |  |
| --- | --- | --- | --- | --- |
|  | **No MP or MR** | **<=1 MP** | **>1 MP** |  |
| **Baseline Age**  **(years)** | 8.7 ±2.0 | 10.3±2.0 | 12.1±2.1 | F=13.8, p<0.0001 |
| **Sex**  **Male**  **Female** | 8/50, 16%  7/58, 12% | 31/50, 62%  37/58, 64% | 11/50, 22%  14/58, 24% | Χ^2^ =0.37, p=0.83 |
| **Ethnicity**  **Asian**  **Non-Asian** | 6/43,14%  9/65,14% | 24/43, 56%  44/65, 68% | 13/43, 30%  12/65, 18% | Χ^2^ =2.12, p=0.35 |
| **Baseline SER**  **(D)** | -2.84±1.4 | -3.22±1.7 | -2.92±1.5 | F=0.55, p=0.58 |
| **Baseline AL**  **(mm)** | 24.80±1.0 | 24.65±0.9 | 24.35±0.91 | F=1.38, p=0.26 |

|  | **2-Year Axial Elongation** | | |  |
| --- | --- | --- | --- | --- |
|  | **<=0mm** | **>0 and <0.3mm** | **>=0.3mm** |  |
| **Baseline Age**  **(years)** | 12.2±1.4 | 11.0±1.9 | 8.7±1.7 | F=35.49, p<0.0001 |
| **Sex**  **Male**  **Female** | 11/50, 22%  8/58, 14% | 15/50, 30%  26/58, 45% | 24/50, 48%  24/58, 41% | Χ^2^=2.85, p=0.24 |
| **Ethnicity**  **Asian**  **Non-Asian** | 8/43, 19%  11/65, 17% | 13/43, 30%  28/65, 43% | 22/43, 51%  26/65, 40% | Χ^2^=1.89, p=0.39 |
| **Baseline SER**  **(D)** | -3.44±1.9 | -3.18±1.7 | -2.90±1.4 | F=0.86, p=0.42 |
| **Baseline AL**  **(mm)** | 24.85±0.8 | 24.74±1.0 | 24.39±0.9 | F=2.51, p=0.09 |

Supplementary material 1. Baseline parameters and participant demographics for 2-year myopia progression and 2-year axial elongation by category. Statistically significant differences between categories were tested by One-Way ANOVA or Chi-Squared. MP= Myopia Progression, MR=Myopia Reduction, SER= Spherical Equivalent Refraction, AL=Axial Length.
